# Supplementary figures and images for: α-Actinin-4 recruits Shp2 into focal adhesions to potentiate ROCK2 activation in podocytes
Source: Life Sci Alliance. 2022 Sep 12;5(11):e202201557. doi: 10.26508/lsa.202201557 (PMC9468603; doi:10.26508/lsa.202201557)

# Source Data\_Figure 1

**Fig 1A**

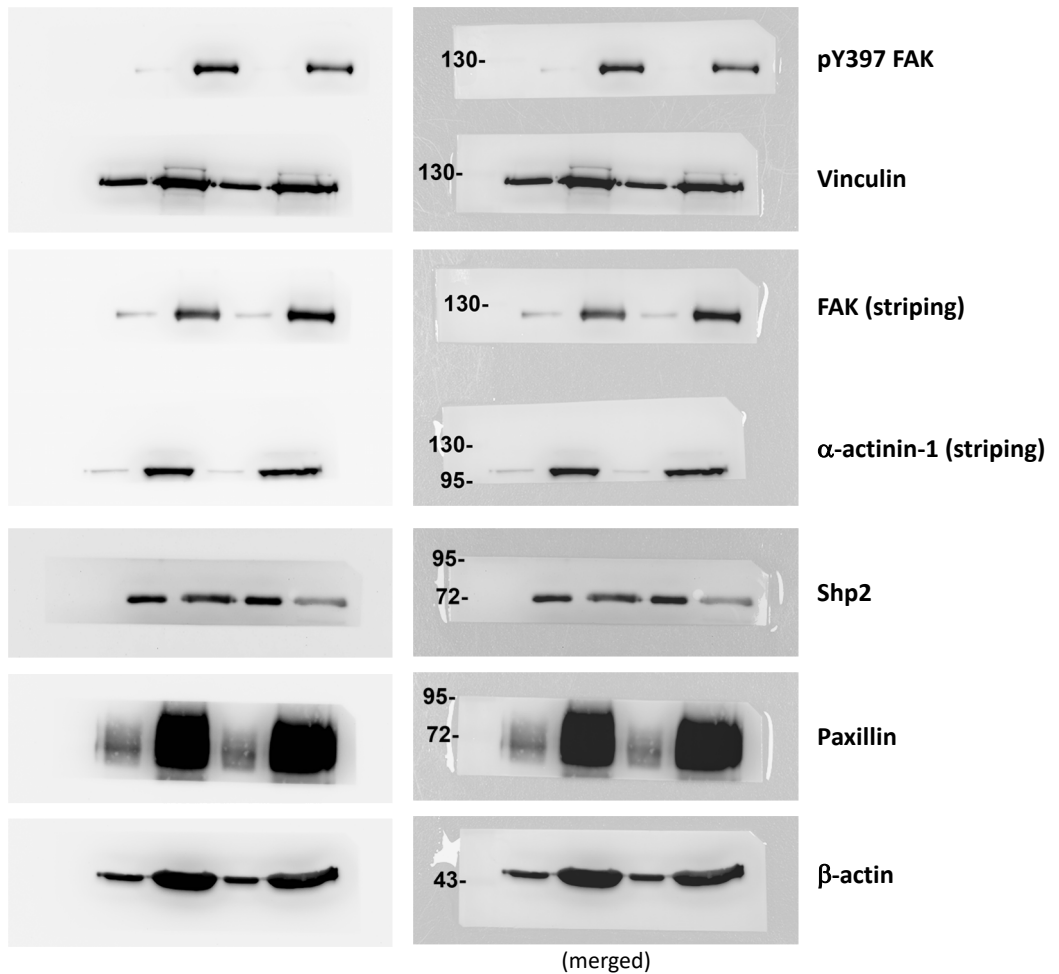

**Fig 1B**

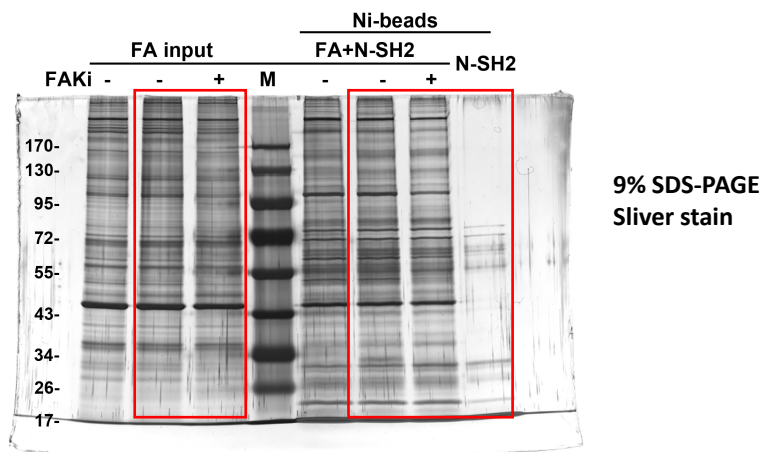

**Fig 1C**

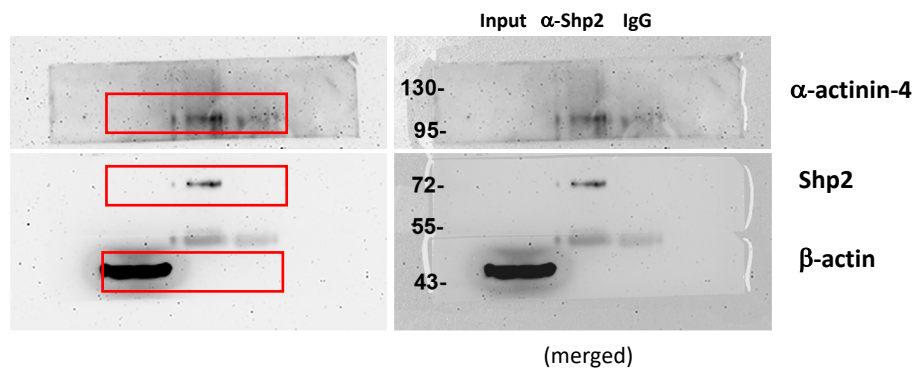

**Fig 1D**

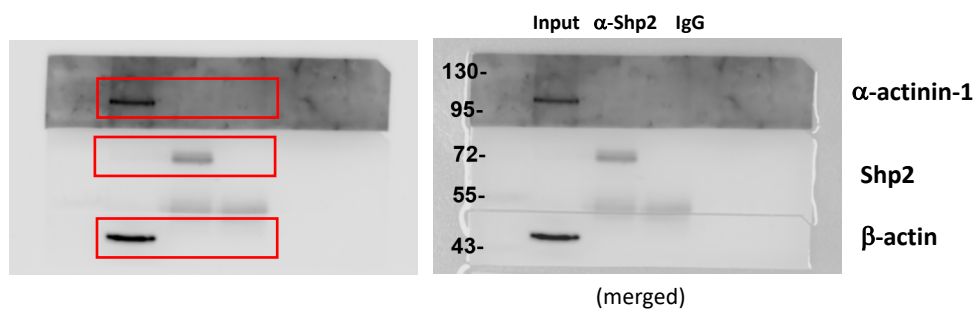

**Fig 1E**

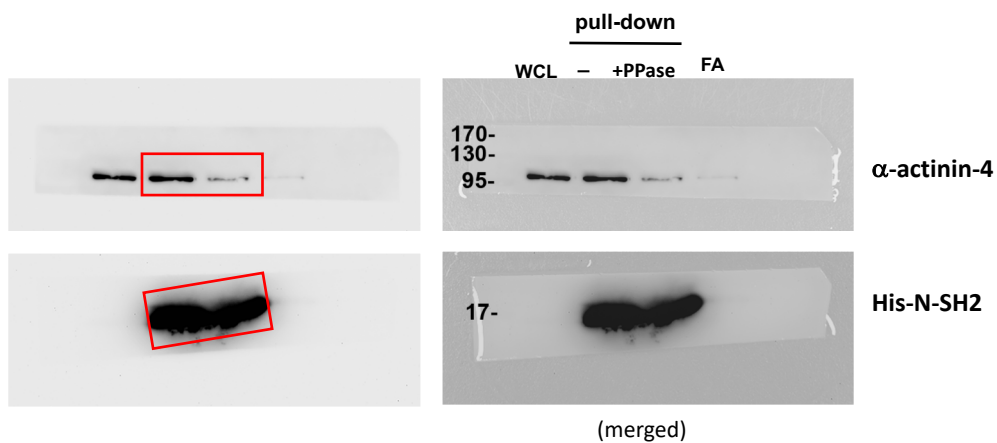

Supplement: Supplementary file 1 [file LSA-2022-01557_SdataF1.pdf]

## Source Data\_Figure 3

Fig 3A

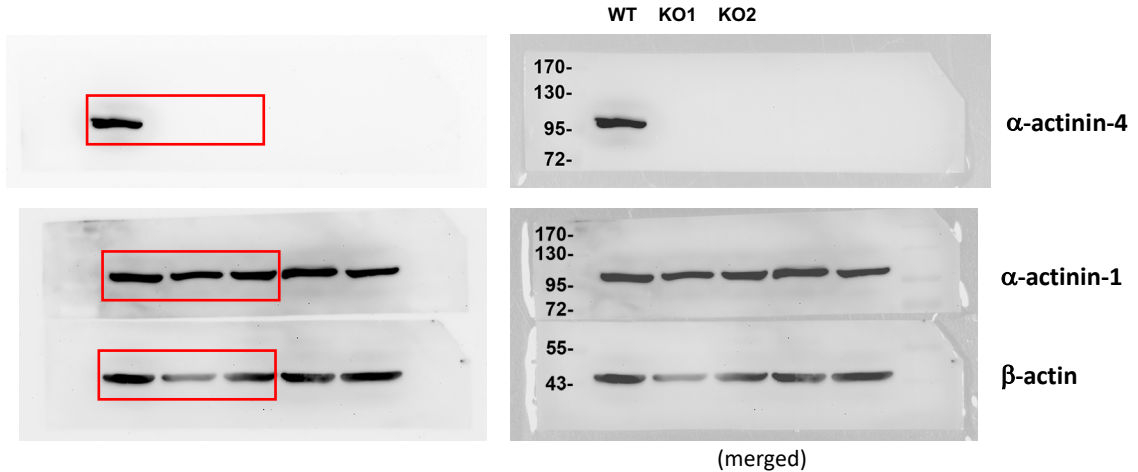

Supplement: Supplementary file 3 [file LSA-2022-01557_SdataF3.pdf]

## Source Data\_Figure 4

Fig 4A

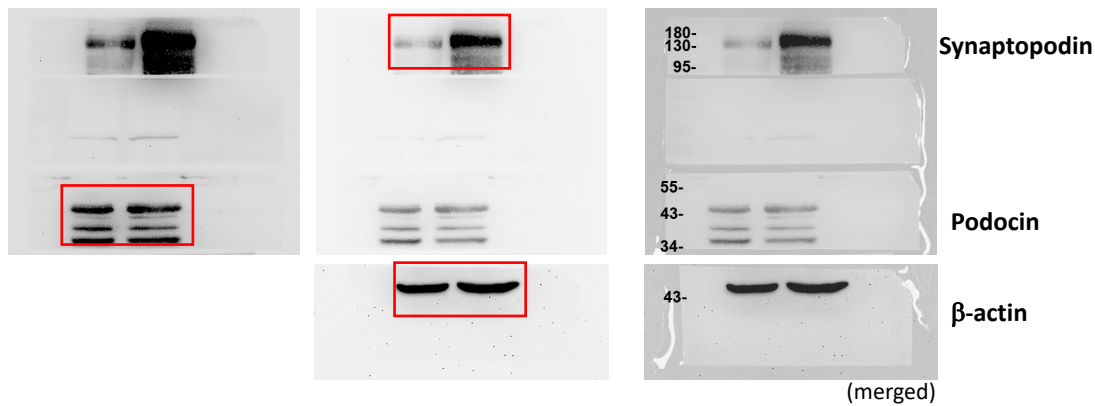

Fig 4C

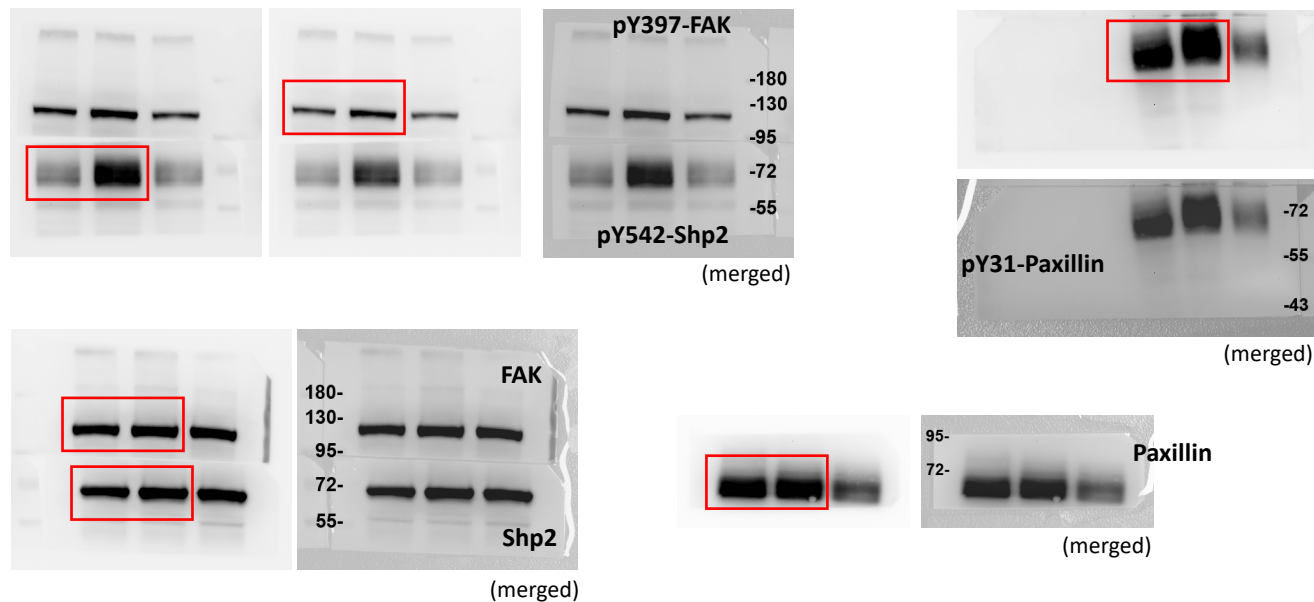

**Fig 4D**

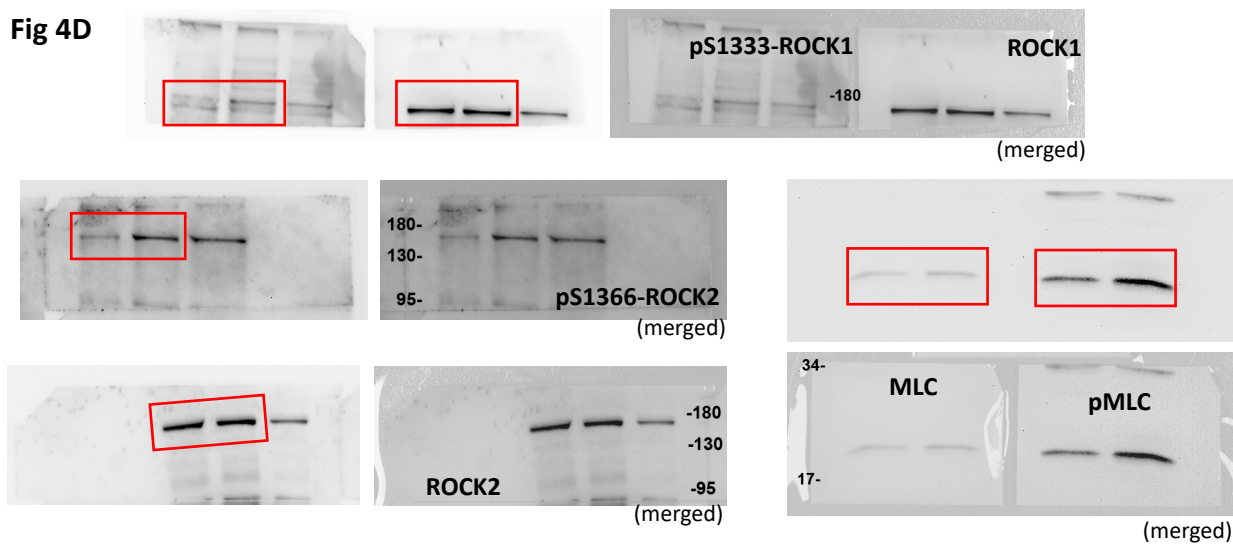

**Fig 4E**

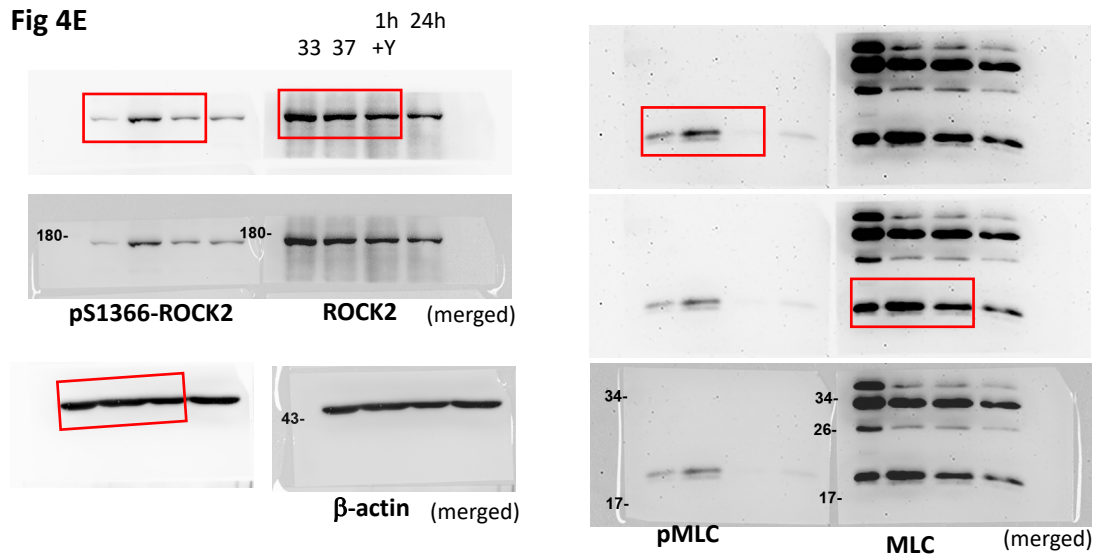

Supplement: Supplementary file 4 [file LSA-2022-01557_SdataF4.pdf]

Source Data\_Figure 5

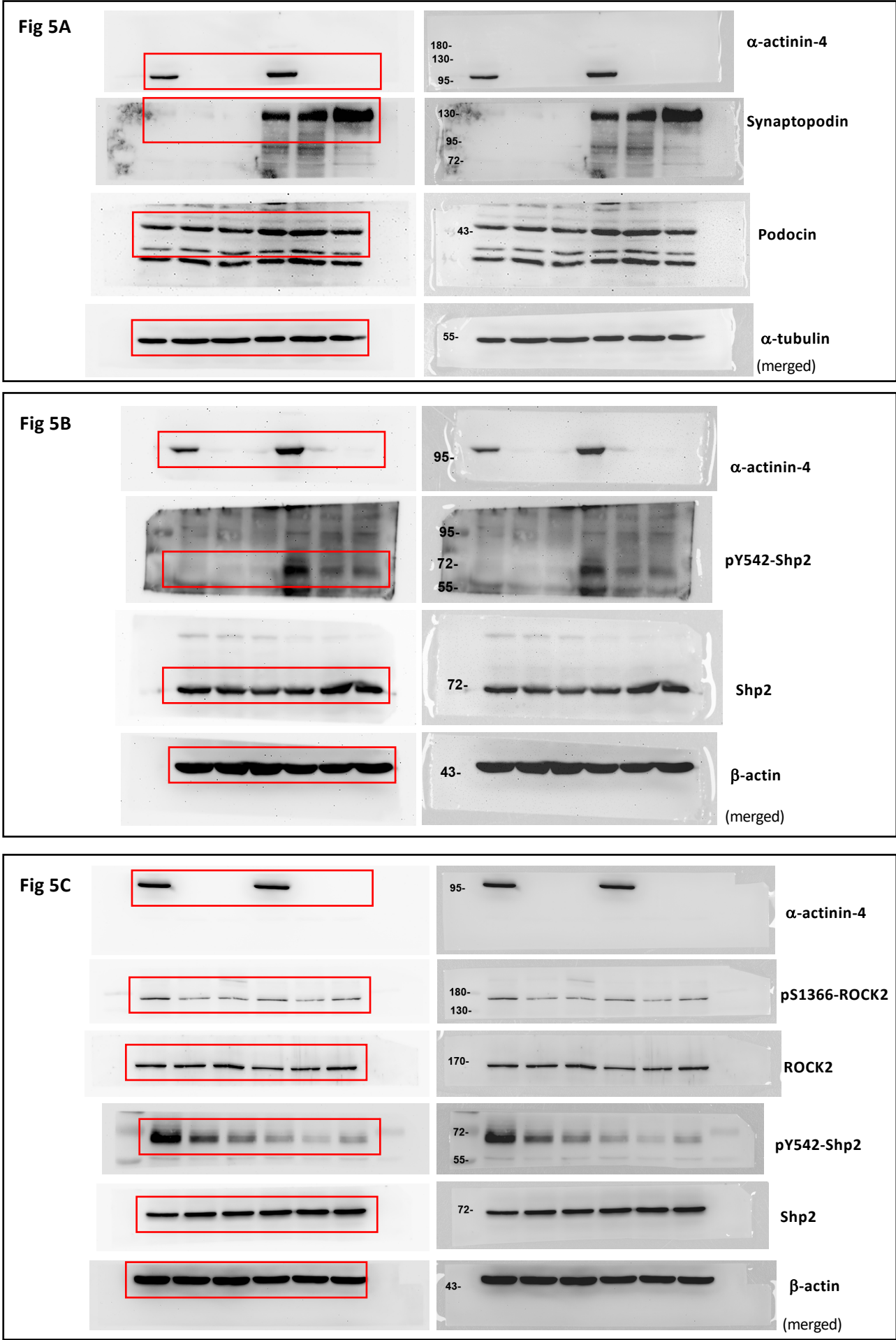

Supplement: Supplementary file 5 [file LSA-2022-01557_SdataF5.pdf]

## Source Data\_Figure 6

Fig 6A

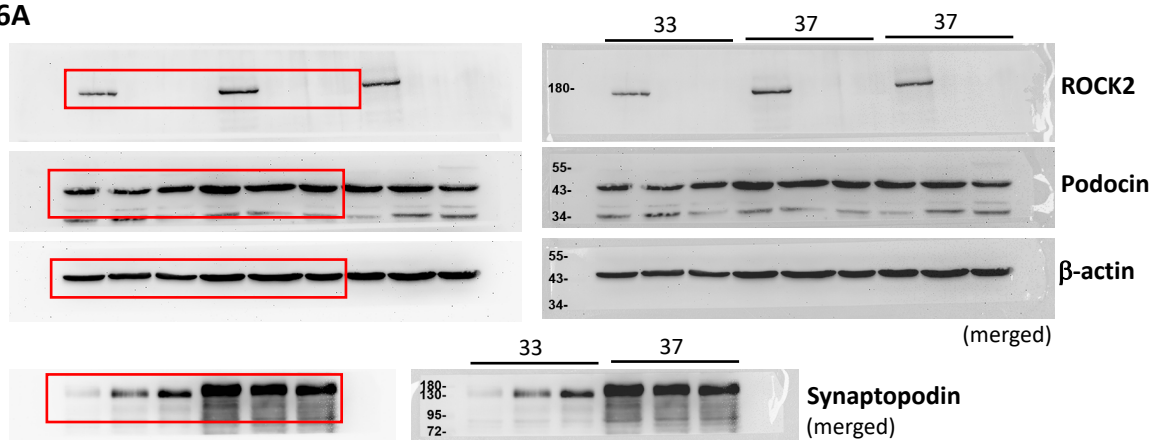

Fig 6B

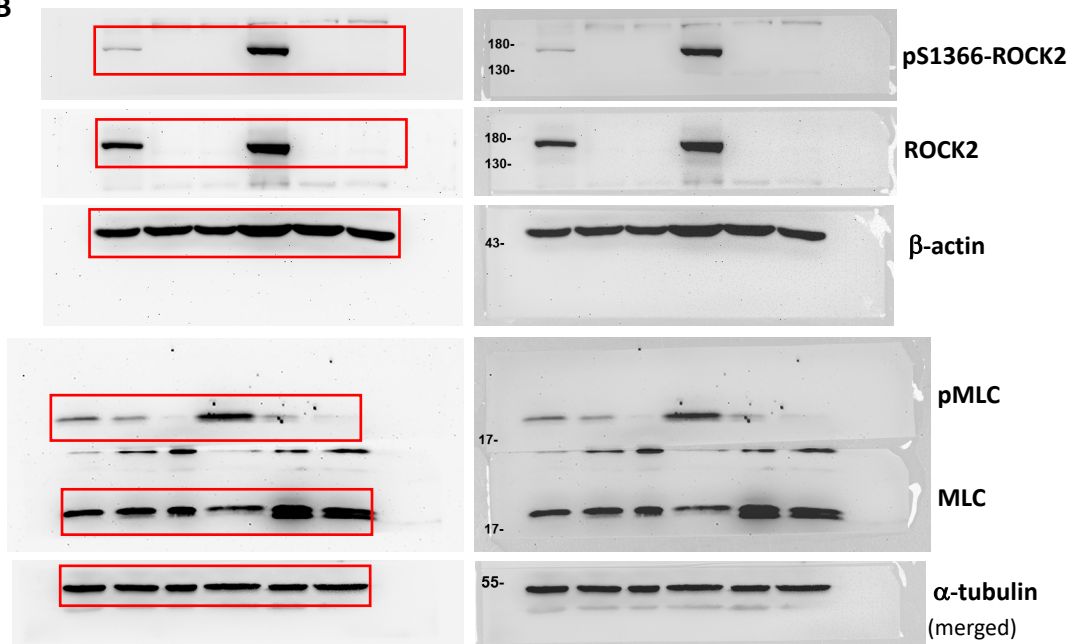

Supplement: Supplementary file 6 [file LSA-2022-01557_SdataF6.pdf]
