# Supplementary material for: α-Actinin-4 recruits Shp2 into focal adhesions to potentiate ROCK2 activation in podocytes
Source: Life Sci Alliance. 2022 Sep 12;5(11):e202201557. doi: 10.26508/lsa.202201557 (PMC9468603; doi:10.26508/lsa.202201557)
Supplement: Supplementary file 2 [file LSA-2022-01557_TableS1.docx]

**Table S1. Mass identification results of FAKi-sensitive N-SH2 interacting proteins in FA fraction**

| Gene | Protein name | MW (Da) | Spectra | Coverage |
| --- | --- | --- | --- | --- |
| Actn4 | alpha-actinin 4 | 104,977 | 116 | 69.0% |
| Ap2b1 | AP-2 complex subunit beta | 104,583 | 12 | 12.9% |
| Actn1 | alpha-actinin 1 | 103,068 | 76 | 42.0% |
| Ctnna1 | Catenin, alpha 1 | 100,106 | 9 | 10.8% |
| Glud1 | Glutamate dehydrogenase 1 | 61,337 | 42 | 44.8% |
| Hspd1 | Heat shock protein 60 | 60,955 | 16 | 24.6% |
| Atp5f1b | ATP synthase subunit beta | 56,300 | 7 | 25.0% |
| Pdlim7 | PDZ and LIM domain protein 7 | 50,119 | 10 | 27.6% |
| Tubb5 | Tubulin beta-5 chain | 49,670 | 7 | 19.8% |
| Vim | Vimentin | 49,193 | 109 | 86.3% |
